# Simultaneous consumption of cellobiose and xylose by *Bacillus coagulans* to circumvent glucose repression and identification of its cellobiose-assimilating operons

**DOI:** 10.1186/s13068-018-1323-5

**Published:** 2018-12-01

**Authors:** Zhaojuan Zheng, Ting Jiang, Lihua Zou, Shuiping Ouyang, Jie Zhou, Xi Lin, Qin He, Limin Wang, Bo Yu, Haijun Xu, Jia Ouyang

**Affiliations:** 1 Jiangsu Co-Innovation Center of Efficient Processing and Utilization of Forest Resources, Nanjing Forestry University, Nanjing, 210037 People’s Republic of China; 2 College of Chemical Engineering, Nanjing Forestry University, Nanjing, 210037 People’s Republic of China; 3 CAS Key Laboratory of Microbial Physiological and Metabolic Engineering, Institute of Microbiology, Chinese Academy of Sciences, Beijing, 100101 People’s Republic of China

**Keywords:** *Bacillus coagulans*, Glucose repression, Cellobiose, Xylose, Cellobiose operon, Phosphoenolpyruvate-dependent phosphotransferase system, *Escherichia coli*

## Abstract

**Background:**

The use of inedible lignocellulosic biomasses for biomanufacturing provides important environmental and economic benefits for society. Efficient co-utilization of lignocellulosic biomass-derived sugars, primarily glucose and xylose, is critical for the viability of lignocellulosic biorefineries. However, the phenomenon of glucose repression prevents co-utilization of both glucose and xylose in cellulosic hydrolysates.

**Results:**

To circumvent glucose repression, co-utilization of cellobiose and xylose by *Bacillus coagulans* NL01 was investigated. During co-fermentation of cellobiose and xylose, *B. coagulans* NL01 simultaneously consumed the sugar mixtures and exhibited an improved lactic acid yield compared with co-fermentation of glucose and xylose. Moreover, the cellobiose metabolism of *B. coagulans* NL01 was investigated for the first time. Based on comparative genomic analysis, two gene clusters that encode two different operons of the cellobiose-specific phosphoenolpyruvate-dependent phosphotransferase system (assigned as CELO1 and CELO2) were identified. For CELO1, five genes were arranged as *celA* (encoding EIIA^cel^), *celB* (encoding EIIB^cel^), *celC* (encoding EIIC^cel^), *pbgl* (encoding 6-phospho-β-glucosidase), and *celR* (encoding a transcriptional regulator), and these genes were found to be ubiquitous in different *B. coagulans* strains. Based on gene knockout results, CELO1 was confirmed to be responsible for the transport and assimilation of cellobiose. For CELO2, the five genes were arranged as *celR*, *celB*, *celA*, *celX* (encoding DUF871 domain-containing protein), and *celC*, and these genes were only found in some *B. coagulans* strains. However, through a comparison of cellobiose fermentation by NL01 and DSM1 that only possess CELO1, it was observed that CELO2 might also play an important role in the utilization of cellobiose in vivo despite the fact that no *pbgl* gene was found. When CELO1 or CELO2 was expressed in *Escherichia coli*, the recombinant strain exhibited distinct cellobiose uptake and consumption.

**Conclusions:**

This study demonstrated the cellobiose-assimilating pathway of *B. coagulans* and provided a new co-utilization strategy of cellobiose and xylose to overcome the obstacles that result from glucose repression in a biorefinery system.

**Electronic supplementary material:**

The online version of this article (10.1186/s13068-018-1323-5) contains supplementary material, which is available to authorized users.

## Introduction

Lignocellulosic biomasses, in particular agricultural and forest residues, represent an attractive resource for replacing fossil fuels. This is because lignocellulosic biomasses are renewable, abundant, and enriched with convertible polysaccharides. Model processes for converting biomass into utilizable fuels, chemicals, and materials are known as lignocellulosic biorefineries. Within a conventional biorefinery process, structural cellulose and hemicellulose are degraded to various monosaccharides after pretreatment and hydrolysis. These resultant monosaccharides can then be further transformed by various microorganisms to produce liquid fuels and platform chemicals [1–4]. The rendered hexose monosaccharides, mainly glucose, mostly hail from cellulose hydrolysis; whereas, the pentoses (mainly xylose if hardwoods and non-woods are processed) are formed from hemicelluloses degraded during pretreatment [5]. However, most microorganisms are unable to ferment xylose, presenting a processing challenge for maximizing the monosaccharides generated by a biorefinery process [6, 7]. Even if a given microorganism can metabolize pentoses, glucose repression is a significant barrier toward effective co-utilization of glucose and xylose mixtures. Specifically, glucose repression causes a sequential or diauxic fermentation of the mixed sugars, resulting in low productivity and, therefore, high processing expenses [7–9]. In general, the phenomenon of glucose repression presents to be a problematic bottleneck for lignocellulosic biorefineries and must be overcome to achieve economic viability.

The common strategy to overcome glucose repression is metabolic engineering of microorganism strains, including deletion of the *ptsG* gene [7], mutation of the *crp* or *ccpA* gene [10], or mutation of the catabolite response element sequence [11]. This partially relieves glucose repression, but co-fermentation of glucose and xylose has been shown to remain inferior relative to single substrate fermentation. However, up to now, investigations into solving the issue of glucose repression by genetic modification have not yielded great breakthroughs. To bypass the problems caused by glucose repression, an alternative strategy is to co-ferment cellobiose, a dimer of glucose, simultaneously with xylose [8]. However, most wild-type microorganisms, such as *Escherichia coli* and yeasts, are unable to ferment cellobiose. As a result, numerous metabolic engineering approaches have been carried out to construct recombinant microorganisms with cellobiose consumption ability. The first strategy was through display of β-glucosidase on the cell surface, which hydrolysed cellobiose into two molecules of glucose outside the cells. However, the co-fermentation process required accurate control of both cellobiose hydrolysis and glucose consumption rates to avoid glucose accumulation [12]. Thereafter, the extracellular hydrolysis of cellobiose was substituted by intercellular hydrolysis [13]. By integration of a cellodextrin transporter, an intracellular β-glucosidase, and the xylose metabolic pathway into *Saccharomyces cerevisiae*, the engineered *S. cerevisiae* was capable of co-fermenting cellobiose and xylose simultaneously [14]. In addition, the role of β-glucosidase can be replaced by cellobiose phosphorylase, which cleaves cellobiose into glucose and glucose-1-phosphate [9, 15].

*Bacillus coagulans* has been demonstrated to be a suitable microorganism for the efficient conversion of biomass-derived monosaccharides into lactic acid, which is an important platform chemical [16, 17]. Many studies reported that *B. coagulans* strains can ferment xylose to lactic acid via the pentose phosphate pathway. In addition, the xylose operon of *B. coagulans* has been analyzed, heterologously expressed, and characterized [18]. Moreover, our previous report showed that *B. coagulans* might also possess an effective cellobiose transportation and metabolism pathway [19]. Therefore, unlike recombinant strains, *B. coagulans* may be an ideal wild-type microorganism with the capability to ferment both cellobiose and xylose. However, co-fermentation of cellobiose and xylose by *B. coagulans* and the pathway of cellobiose metabolism in *B. coagulans* have not yet been studied.

With that, we aimed to evaluate co-utilization of cellobiose and xylose by *B. coagulans* for lactic acid production and to identify the genes responsible for cellobiose catabolism in *B. coagulans*. Firstly, the ability of *B. coagulans* to convert cellobiose into lactic acid was investigated. Next, the fermentation performance of *B. coagulans* was examined using mixture of cellobiose and xylose. Based on these results, we intended to then probe for the cellobiose operons present in *B. coagulans*. The goal of this study was to advance the utilization of cellobiose and xylose mixtures in a biorefinery setting with hopes that *B. coagulans* will be recognized as a strong candidate for improving the efficiency and overall economics of biorefinery processes.

## Materials and methods

### Bacterial strains and media

All strains, plasmids, and primers used in this study are listed in Table 1. *B. coagulans* strains were maintained on a GYC agar slant containing (g/L): glucose 20, yeast extract 1, corn steep liquor powder 2.5, NH_4_Cl 1, MgSO_4_·7H_2_O 0.2, and CaCO_3_ 10. The pH was adjusted to 7.2 using NaOH before adding CaCO_3_. The slant was incubated at 50 °C for 14 h and stored at 4 °C. For genetic engineering, *B. coagulans* ATCC 7050 was grown in BC medium at 45 °C and 120 rpm [20].

**Table 1:** Strains, plasmids and primers used in this study

Designation	Relevant characteristics	Source or reference
Strains		
*B. coagulans* NL01	Undomesticated wild strain	Laboratory preservation
*B. coagulans* ATCC 7050	Undomesticated wild strain	American Type Culture Collection
*B. coagulans* XZL4	Undomesticated wild strain	Professor Ping Xu^a^
*B. coagulans* ATCC 7050 (Δ*celR*)	*celR* deletion strain of *B. coagulans* ATCC 7050	This study
*L. lactis* MG1363	Host for gene cloning	Laboratory preservation
*E. coli* BL21(DE3)	Host for protein expression	Invitrogen
*E. coli* BL21 (pET-*celo1*)	*E. coli* BL21(DE3) harboring the plasmid pET-*celo1*	This study
*E. coli* BL21 (pET-*celo2*)	*E. coli* BL21(DE3) harboring the plasmid pET-*celo2*	This study
*E. coli* BL21 (pETDuet-1)	*E. coli* BL21(DE3) harboring the plasmid pETDuet-1	This study
Plasmids		
pMH77	pSH71 replication containing temperature-sensitive vector, Cm^r^	Professor Oscar P. Kuipers^b^
pMD-19T	Gene cloning vector, Amp^r^	TaKaRa
19T-*celR*1	pMD-19T containing the flanking regions of *celR*	This study
pMH77-*celR*1	pMH77 containing the flanking regions of *celR*	This study
pETDuet-1	Protein expression vector, Amp^r^	Novagen
pET-*celo1*	pETDuet-1 containing the CELO1 from *B. coagulans* NL01	This study
pET-*celo2*	pETDuet-1 containing the CELO2 from *B. coagulans* NL01	This study
Primers	Sequences (5′ → 3′) and properties	
*celR*1up.f	GATCCGGAATTCCCACACAAAGGGAAATTACGTAAGCCAC (*Eco*RI)	This study
*celR*1up.r	GTAAATGGCGGCGGCACTGAGCTCATGCCAACAACTCCCTTTTATCATTG	This study
*celR*1down.f	CAATGATAAAAGGGAGTTGTTGGCATGAGCTCAGTGCCGCCGCCATTTAC	This study
*celR*1down.r	CATCCGCTCGAGCAATATCATCCAGTTTCACCATATTCGG (*Xho*I)	This study
*celo*1.f	AGATCTCTTGAACATAGAAGAAATCAGTTTTC (*Bgl*II)	This study
*celo*1.r	GGTACCTTATTTTATACATGCCATTAACTCAT (*Kpn*I)	This study
*celo*2.f	GAGCTCGTTGATTTCGAATCGGCAAAAAC (*Sac*I)	This study
*celo*2.r	CTCGAGTTAAGCAATGCTATCCTTCGTCGT (*Xho*I)	This study

^a^From State Key Laboratory of Microbial Metabolism, Shanghai Jiao Tong University, People’s Republic of China

^b^From Groningen Biomolecular Sciences and Biotechnology Institute, Department of Genetics, University of Groningen, Netherlands

*Escherichia coli* strains were cultivated in Luria Broth (LB) medium, which contained 10 g/L tryptone, 5 g/L yeast extract, and 10 g/L NaCl. When needed, 100 mg/L ampicillin was added for plasmid maintenance.

### Fermentation experiments of *B. coagulans* strains

A loop of cells collected from a fully grown slant was inoculated into a 150-mL flask containing 50 mL of GYC liquid medium as a seed culture. The culture was incubated for 14 h at 50 °C with 150 rpm and then inoculated with a 10% (v/v) inocula into Erlenmeyer flasks with for lactic acid production. Fermentations were conducted in 250-mL Erlenmeyer flasks containing 100 mL of medium with shaking at 50 °C and 150 rpm on a rotary shaker. The medium for cellobiose fermentation contained the following (g/L): various amount of cellobiose (20–120), yeast extract 2.5, corn syrup powder 1.2, MgSO_4_·7H_2_O 0.4, (NH_4_)_2_SO_4_ 3, KH_2_PO_4_ 0.22, MnSO_4_·H_2_O 0.03, FeSO_4_·H_2_O 0.03, and CaCO_3_ at 1/2 of the added sugar by weight. For the glucose/xylose and cellobiose/xylose co-utilization experiments, the sugars were replaced by 5 g/L glucose and 5 g/L xylose, 5 g/L cellobiose and 5 g/L xylose, 40 g/L glucose and 40 g/L xylose, and 40 g/L cellobiose and 40 g/L xylose, respectively. Samples were taken periodically to determine the concentrations of residual sugars and the produced lactic acid. All experiments were performed in triplicate.

### RNA isolation, cDNA generation, and RT-PCR

Total RNA was isolated from the *B. coagulans* NL01 cells grown to the exponential phase under inducing conditions (with cellobiose as the sole carbon source), using a Qiagen RNeasy total RNA kit (Qiagen, Germany). The integrity of the RNA was guaranteed by electrophoresis of total RNA in a 1.5% agarose gel. The gDNA was erased in advance, and cDNA was generated by PrimeScript™ RT reagent kit with a gDNA eraser (TaKaRa, China). Reverse transcription PCR (RT-PCR) was performed in accordance with the standard procedures using 1 μM each specific primer (see Additional file 1: Table S1). The genomic DNA of *B. coagulans* NL01 was used as a positive control.

### Gene knockout procedure of *B. coagulans* ATCC 7050

The *celR* gene knockout of *B. coagulans* ATCC 7050 was performed using plasmid pMH77. All procedures were conducted according to a previous Ref. [21]. Genomic DNA of *B. coagulans* ATCC 7050 was extracted using the Wizard Genomic DNA Purification Kit (Promega, Madison, WI, USA). The flanking regions of the *celR* gene were amplified from *B. coagulans* ATCC 7050 genomic DNA using the primers *celR*1up.f and *celR*1up.r (upstream, 1000 bp) and *celR*1down.f and *celR*1down.r (downstream, 1000 bp), respectively. After gel purification, overlap extension PCR was performed, wherein the upstream and downstream regions were fused using the primers *celR*1up.f and *celR*1down.r. The resulting PCR product was gel purified and ligated into pMD-19T to form a new plasmid, 19T-*celR*1. The plasmid 19T-*celR*1 was digested by *Eco*RI and *Xho*I and cloned into the corresponding sites of pMH77 to obtain another plasmid, pMH77-*celR*1. The plasmid pMH77-*celR*1 was first transformed into *Lactococcus lactis* MG1363. The extracted plasmid from *L. lactis* MG1363 was then as transformed into *B. coagulans* ATCC 7050 by electroporation.

A colony of *B. coagulans* ATCC 7050 harboring pMH77-*celR*1 was cultured at 45 °C for 12 h and then shifted to 60 °C for further incubation for 12 h. The diluted cells were plated on a BC plate supplemented with 7 mg/L chloramphenicol and cultured at 60 °C to select the first crossover cells. The right single crossover colony was incubated in BC liquid broth without chloramphenicol overnight at 45 °C and then spread onto BC plates without chloramphenicol. Plenty of colonies were sequentially spread onto BC plates with and without chloramphenicol and incubated overnight at 45 °C. The colony that grew on BC plates without chloramphenicol, but did not grow on BC plates with chloramphenicol, was the double crossover cell. The resulting deletion mutant was designated as *B. coagulans* ATCC 7050 (Δ*celR*). All the constructed strains were validated by PCR and DNA sequencing.

### Utilization of cellobiose by recombinant *E. coli* strains

The cellobiose operons of *B. coagulans* NL01 were amplified from the genomic DNA with primers *celo*1.f/*celo*1.r and *celo*2.f/*celo*2.r. The PCR product was cloned into the expression plasmid pETDuet-1 between unique *Bgl*II/*Kpn*I and *Sac*I/*Xho*I sites to yield the recombinant plasmid pET-*celo1* and pET-*celo2*, respectively. The plasmids pET-*celo1* and pET-*celo2* were transformed into *E. coli* BL21(DE3) to construct the recombinant *E. coli* BL21 (pET-*celo1*) and *E. coli* BL21 (pET-*celo2*).

The resulting *E. coli* BL21 (pET-*celo1*), *E. coli* BL21 (pET-*celo2*) and a control with the same host bearing an empty plasmid were cultured in LB medium overnight. This culture was inoculated with 5% into 30-mL LB medium in the presence of 10 g/L cellobiose, 100 mg/L ampicillin, and 0.2 mM IPTG. The cells were further cultured at 37 °C and 200 rpm. The samples were collected periodically for determination of the cell density and the residual cellobiose. All experiments were performed in triplicate.

### Analytic methods

Glucose, xylose, cellobiose, and lactic acid were analyzed using an Agilent HPLC 1260 system with a Bio-Rad Aminex HPX-87H column (300 × 7.8 mm) and a refractive index detector. The mobile phase was 5 mM H_2_SO_4_ at a flow rate of 0.6 mL/min, and the column temperature was maintained at 55 °C [6, 19]. The cell density of *E. coli* was estimated by determining the optical density at 600 nm.

Productivity (g/L/h) was calculated as the concentration of lactic acid produced per liter divided by the fermentation time (h). Lactic acid yield was calculated as the percentage of measured lactic acid relative to the theoretical amount of lactic acid producible. The theoretical amount was calculated as follows: glucose (1 g lactic acid/g glucose), xylose (1 g lactic acid/g xylose) and cellobiose (1.05 g lactic acid/g cellobiose) [1].

The activity of 6-phospho-β-glucosidase was determined using phosphorylated *p*-nitrophenyl-β-d-glucopyranoside-6-phosphate (pNPβG6P) as a substrate. The assay was carried out with a total volume of 1 mL, containing 50 mM PBS (pH 7), 1 mM pNPβG6P and 50 μL of suitably diluted crude enzyme. The reaction was incubated at 50 °C for 2 min and terminated by the addition of 500 μL of 1 M Na_2_CO_3_. The released *p*-nitrophenol (pNP) was determined by the absorbance at 410 nm. One unit of 6-phospho-β-glucosidase was defined as the amount of enzyme catalyzing the appearance of 1 μmol of pNP per min under experimental conditions.

## Results and discussion

### Efficient cellobiose utilization by *B. coagulans* NL01

As the basis for the current work, our previous studies found that *B. coagulans* strain NL01 had the ability to use cellobiose as carbon source to produce lactic acid [16, 19]. In this study, we first carried out the pure sugar fermentation at a concentration of 40 g/L glucose, cellobiose or xylose. The results showed that all three substrates were consumed quickly, and the lactic acid yields from glucose, cellobiose, and xylose were 94.1%, 91.1%, and 89.5%, respectively. These demonstrated that *B. coagulans* NL01 can efficiently utilize the lignocellulosic biomass-derived sugars.

**Fig. 1:**
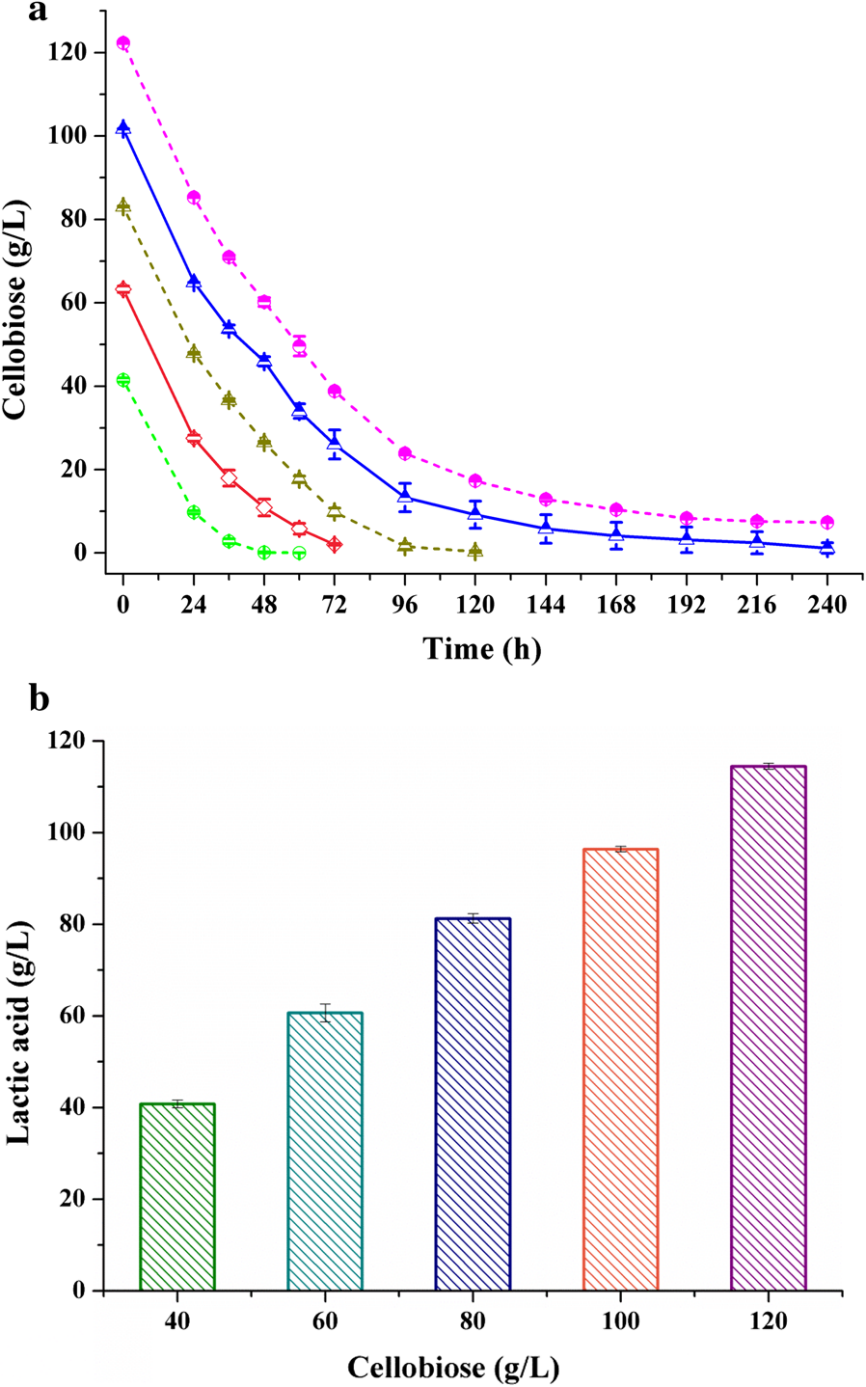
Fermentation of cellobiose by *B. coagulans* NL01. **a** Time course of cellobiose utilization under different initial concentrations. Green, 40 g/L; Pink, 60 g/L; Dark yellow, 80 g/L; Blue, 100 g/L; Magenta, 120 g/L. **b** Effect of cellobiose concentration on lactic acid production. The maximum concentration of lactic acid was obtained after 60, 72, 120, 240, and 240 h, respectively. Fermentations were carried out in 250-mL Erlenmeyer flasks containing 100-mL medium at 50 °C and 150 rpm on a rotary shaker. The medium contained the following (g/L): various amounts of cellobiose (40–120), yeast extract 2.5, corn syrup powder 1.2, MgSO_4_·7H_2_O 0.4, (NH_4_)_2_SO_4_ 3, KH_2_PO_4_ 0.22, MnSO_4_·H_2_O 0.03, FeSO_4_·H_2_O 0.03, and CaCO_3_ at 1/2 of the added sugar by weight. Values are the average of three independent experiments ± SD

To evaluate the maximum potential of *B. coagulans* NL01 for cellobiose utilization, fermentation was conducted with different initial concentrations of cellobiose, ranging from 40 g/L to 120 g/L. As shown in Fig. 1a, *B. coagulans* NL01 was capable of fermenting cellobiose efficiently regardless of its initial concentration. With the increased initial cellobiose concentration, the maximum concentration of lactic acid was raised from 40.8 to 114.5 g/L, and the yield varied from 91.1 to  94.8% (Fig. 1b). It should be noted that no fermentation byproducts, such as acetic acid or ethanol, were found present in the fermentation broth. Several other *B. coagulans* strains have been reported to have the capacity to ferment cellobiose for lactic acid production. For example, *B. coagulans* 36D1 was applied to production of lactic acid from cellulose through simultaneous saccharification and fermentation (SSF) in a fed-batch mode, which obtained a yield rate of approximately 88% [22]. *B. coagulans* LA204 produced lactic acid with a yield of 85.5% from 50 g/L cellobiose [23]. *B. coagulans* IPE22 can also use cellobiose; however, the fermentation performance of this strain under high cellobiose concentrations was not reported [24]. The best fermentation was performed by *B. coagulans* WCP10-4, which produced 196.3 g/L lactic acid from 200 g/L cellobiose in a 2-L fermentor [25]. Although previous papers have reported cellobiose fermentation, they did not focus on simultaneous consumption of cellobiose and xylose. Moreover, these studies did not investigate the genes responsible for cellobiose metabolism.

### Comparison of glucose/xylose and cellobiose/xylose co-utilization

As we introduced, xylose utilization by microorganisms is inhibited when glucose accumulates massively during SSF of lignocellulosic biomass. Interestingly, many recombinant strains demonstrated that cellobiose and xylose can assimilate simultaneously. However, few similar experiments have been conducted in wild strains. To demonstrate the superiority of cellobiose/xylose co-utilization in *B. coagulans* NL01, co-fermentation of glucose/xylose and cellobiose/xylose mixtures was carried out and compared.

**Fig. 2:**
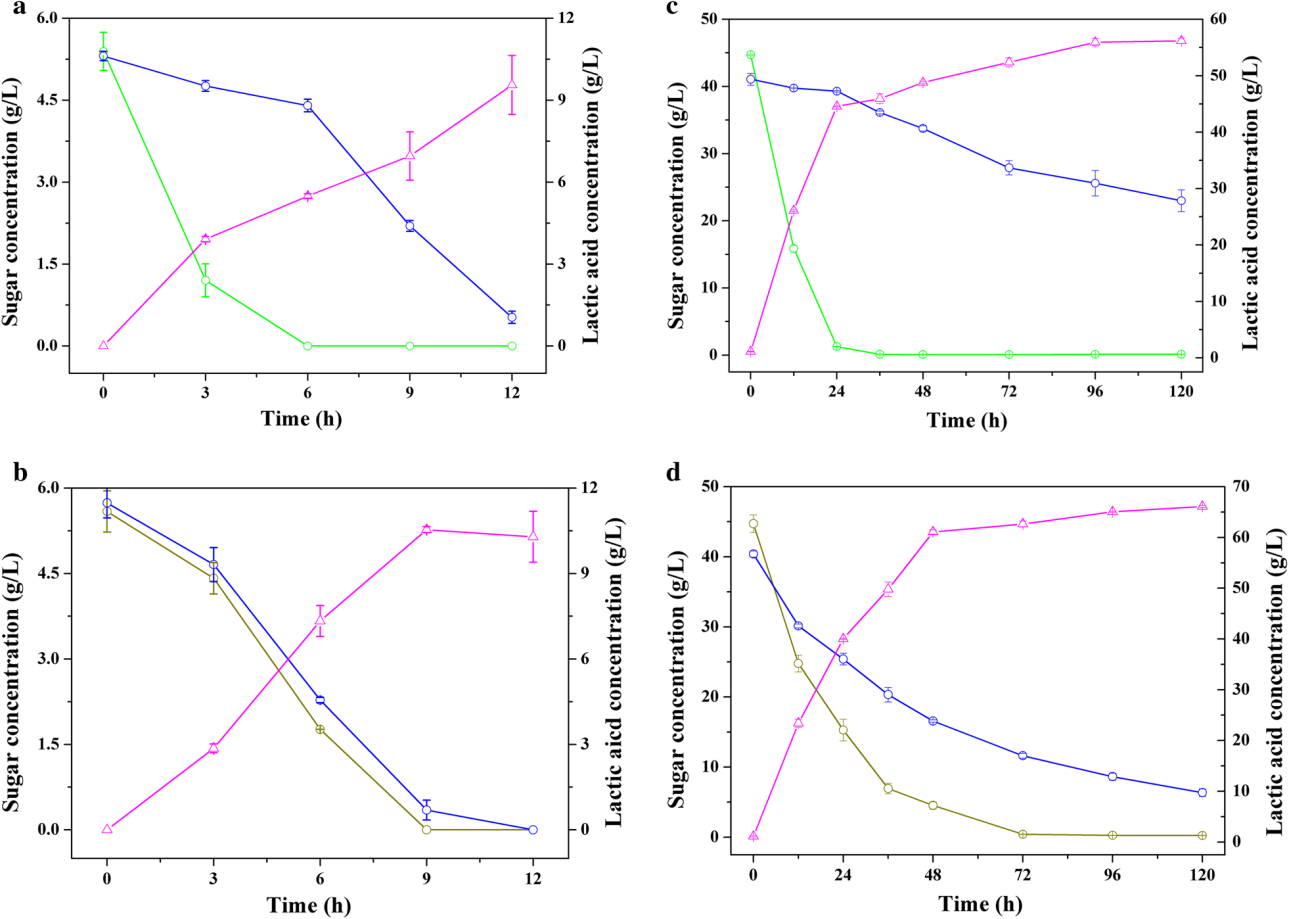
Co-fermentation of glucose/xylose or cellobiose/xylose mixtures for lactic acid production by *B. coagulans* NL01. Green, glucose; Dark yellow, cellobiose; Blue, xylose; Magenta, lactic acid. Fermentations were carried out in 250-mL Erlenmeyer flasks containing 100-mL medium at 50 °C and 150 rpm on a rotary shaker. The medium contained the following (g/L): glucose 5 and xylose 5 (**a**)/cellobiose 5 and xylose 5 (**b**)/glucose 40 and xylose 40 (**c**)/cellobiose 40 and xylose 40 (**d**), yeast extract 2.5, corn syrup powder 1.2, MgSO_4_·7H_2_O 0.4, (NH_4_)_2_SO_4_ 3, KH_2_PO_4_ 0.22, MnSO_4_·H_2_O 0.03, FeSO_4_·H_2_O 0.03, and CaCO_3_ at 1/2 of the added sugar by weight. Values are the average of three independent experiments ± SD

Figure 2 shows both sugar consumption and lactic acid production by *B. coagulans* NL01 at a total sugar concentration of approximately 10 g/L (Fig. 2a, b) and 80 g/L (Fig. 2c, d). During co-fermentation of glucose and xylose, as expected, xylose utilization was retarded during the early phases of fermentation. When glucose was depleted, the xylose uptake rate was markedly hastened. This observation indicated that glucose repression did occur. Figure 2a shows that the final lactic acid concentration was 9.6 g/L at a 12-h fermentation, representing 94.9% lactic acid yield. However, there remained some unutilized xylose in the fermentation medium at the conclusion of fermentation time. With the concentrations of sugars increasing, xylose consumption would be more inhibited. Moving on to the 80 g/L fermentation experiment, after a 120-h fermentation, approximately 23.0 g/L xylose remained (Fig. 2c). For the fermentation containing cellobiose and xylose, *B. coagulans* NL01 consumed both sugars simultaneously during fermentation (Fig. 2b, d). It was also be observed that both sugars were almost completely consumed, translating to a high lactic acid concentration [10.5 g/L at 9 h fermentation (Fig. 2b)]. In contrast, only 7 g/L lactic acid was produced within 9 h when co-fermentation involved 5 g/L glucose and 5 g/L xylose (Fig. 2a). As for co-fermentation of 40 g/L cellobiose and 40 g/L xylose, approximately 66.1 g/L lactic acid was produced at the fermentation end-point. The residual xylose was 6.4 g/L, which was much lower than that in Fig. 2c, d). The observation of incomplete xylose consumption could be explained by a lessened ability of *B. coagulans* NL01 regarding fermentation of xylose relative to fermentation of cellobiose. In fact, when using 80 g/L cellobiose or xylose as the substrate, cellobiose was consumed completely (Fig. 1a), whereas xylose remained in surplus (above 5 g/L) [26]. In all, the results of Fig. 2 indicate that *B. coagulans* NL01 was able to co-ferment cellobiose and xylose rapidly and efficiently (despite residual xylose).

### Two different cellobiose metabolic operons found in *B. coagulans* NL01

So far, cellobiose metabolism in *B. coagulans* has not been reported. The excellent fermentation performance using cellobiose led us to further investigate the specific catabolic pathways in *B. coagulans* NL01 that provide the demonstrated superiority. At present, two different pathways of cellobiose degradation are known in microorganisms: hydrolysis (depending on β-glucosidase) and phosphorolysis (depending on cellobiose phosphorylase). These pathways have been elucidated as well as exploited in various instances of metabolic engineering [9, 15, 27]. Therefore, we have attempted to determine the activities of both β-glucosidase and cellobiose phosphorylase enzymes in *B. coagulans* NL01. However, no β-glucosidase or cellobiose phosphorylase activity was detected in any subcellular locations of *B. coagulans* NL01 in our studies.

Currently, numerous *B. coagulans* strains have undergone genomic sequencing and annotation, including *B. coagulans* NL01. In agreement with our previous discussion, no protein predicted as β-glucosidase or cellobiose phosphorylase could be found in the *B. coagulans* NL01 genome. Instead, a protein annotated as a putative 6-phospho-β-glucosidase (Genbank accession No. WP_046721809) was searched, which belongs to glycosyl hydrolase family 1. Previous studies reported that the β-1,4-glycosidic bond can be broken down by phosphoglycosyl hydrolases, such as 6-phospho-β-glucosidase, by the aid of the phosphoenolpyruvate-dependent phosphotransferase system (PEP-PTS) [28]. Therefore, we next attempted to determine 6-phospho-β-glucosidase enzymatic activity in *B. coagulans* NL01. When *B. coagulans* NL01 was cultivated using glucose, xylose or cellobiose as the sole carbon source, 6-phospho-β-glucosidase activity was detected in the presence of cellobiose (0.29 U/mg), but not in the presence of glucose or xylose. This observation indicated that 6-phospho-β-glucosidase may play an essential role in cellobiose metabolism and that expression of this enzyme required cellobiose for induction.

PEP-PTS is usually composed of EI, HPr and EII(s). EI and HPr are the general cytoplasmic PTS components. In contrast, EII(s) are substrate-specific permeases that consist of three or four domains IIA, IIB, IIC and IID (present only in a few families). They can be encoded by a single or multiple genes organized in an operon [28–30]. For catabolism of cellobiose, the cellobiose operon has been reported in some lactic acid bacteria, such as *Streptococcus mutans* [31] and *Lactobacillus plantarum* [32]. The genetic organization of cellobiose operons from different strains was quite different, but all included genes at least for 6-phospho-β-glucosidase (*pbgl*), a transcriptional regulator (*celR*), IIA (*celA*), IIB (*celB*), and IIC (*celC*).

**Fig. 3:**
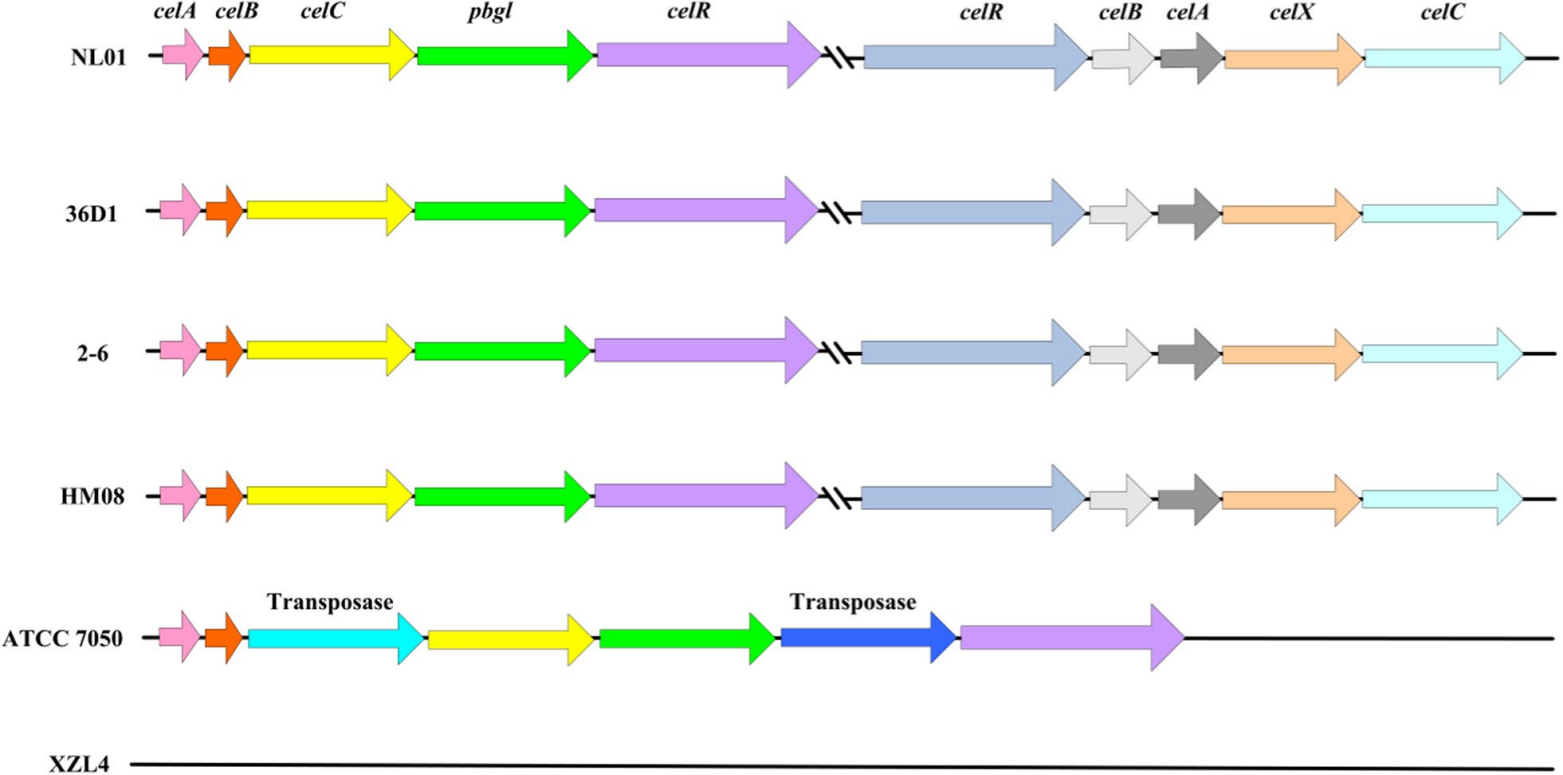
Organization of cellobiose operons in different *B. coagulans* strains. Arrows indicate the direction of gene translation

Up to now, no data related to a cellobiose operon in *B. coagulans* have been disclosed. Therefore, we sought to reveal all relevant components of the cellobiose operon in the *B. coagulans* NL01 genome. Figure 3 shows that this strain possesses two different potential cellobiose operons (assigned as CELO1 and CELO2) and that they were found to be not adjacent to one another. Each operon was composed of five genes and arranged in the same direction. For CELO1, the five genes were arranged as *celA*, *celB*, *celC*, *pbgl*, and *celR*. For CELO2, unexpectedly, no *pbgl* gene was found. Instead, a gene annotated as a DUF871 domain-containing protein (hereafter denoted as *celX*) was identified (DUF represents domains of unknown function). They were organized as *celR*, *celB*, *celA*, *celX*, and *celC*. Moreover, there was no sequence identity between the corresponding components of CELO1 and CELO2.

**Fig. 4:**
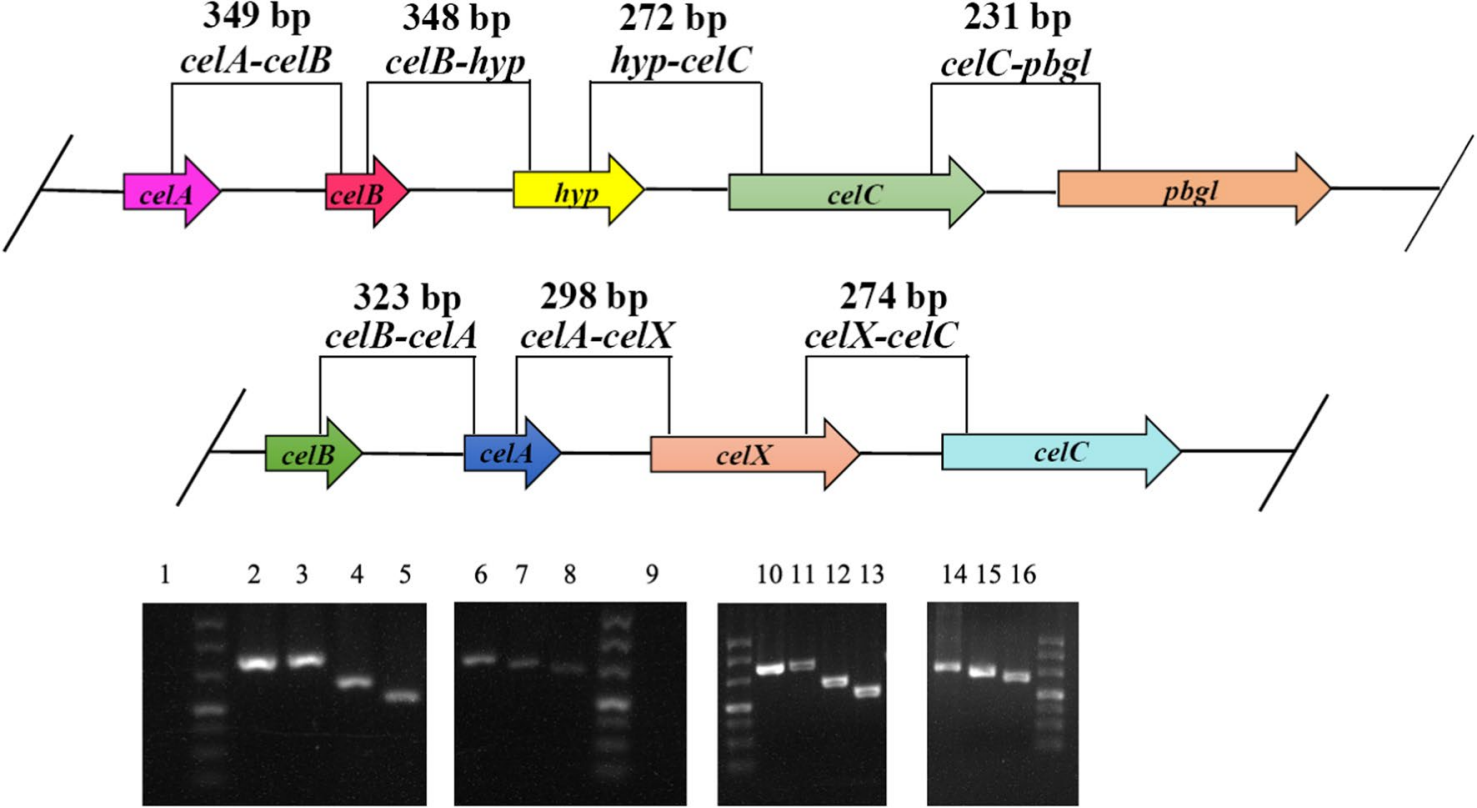
Identification of the co-transcription of structural genes in the CELO1 and CELO2 operon using RT-PCR. cDNA was isolated and RT-PCR was performed as described in the text (for lane 2–8). The RNA and genomic DNA of *B. coagulans* NL01 was used as negative (for lane 1 and 9) and positive control (for lane 10–16), respectively. The amplified overlapping regions spanned *celA*–*celB* (lane 1, 2, and 10), *celB*–*hyp* (lane 3 and 11), *hyp*–*celC* (lane 4 and 12), *celC*–*pbgl* (lane 5 and 13), *celB*–*celA* (lane 6, 9, and 14), *celA*–*celX* (lane 7 and 15), *celX*–*celC* (lane 8 and 16)

The co-transcription of structural genes in the CELO1 and CELO2 was certified by an RT-PCR approach. The cDNA product generated from total RNA extracted from cells induced by cellobiose was used as template in RT-PCR to examine the amplification of overlapping regions spanning each two adjacent genes. For CELO1, because the length from *cleB* to *celC* was longer than other intervals and a hypothetic gene (*hyp*) was predicted between them, the amplified overlapping region spanning *cleB*–*celC* was substituted by regions spanning *cleB*–*hyp* and *hyp*–*celC* (Fig. 4). Figure 4 shows that only a single PCR product was obtained from each amplification reaction, and the size of each fragment from cDNA was consistent with those from genomic DNA. These results proved that both of the gene clusters are co-transcribed as an operon.

### The role of CELO1 and CELO2 in cellobiose utilization

To investigate whether other *B. coagulans* strains possess similar cellobiose operons, another five typical strains that had been sequenced and reported were selected. The chosen strains were *B. coagulans* 36D1, 2-6, HM-08, ATCC 7050 and XZL4. Figure 3 indicates that the cellobiose operons were ubiquitous in most *B. coagulans* strains. For strains NL01, 36D1, 2-6, and HM-08, all of them had two cellobiose operons, and comparisons between them revealed a high degree of synteny. For strain ATCC 7050, only the cellobiose operon CELO1 was found, and it was interspersed with two transposase genes. Strain XZL4 was the sole exception which had no cellobiose operon.

**Fig. 5:**
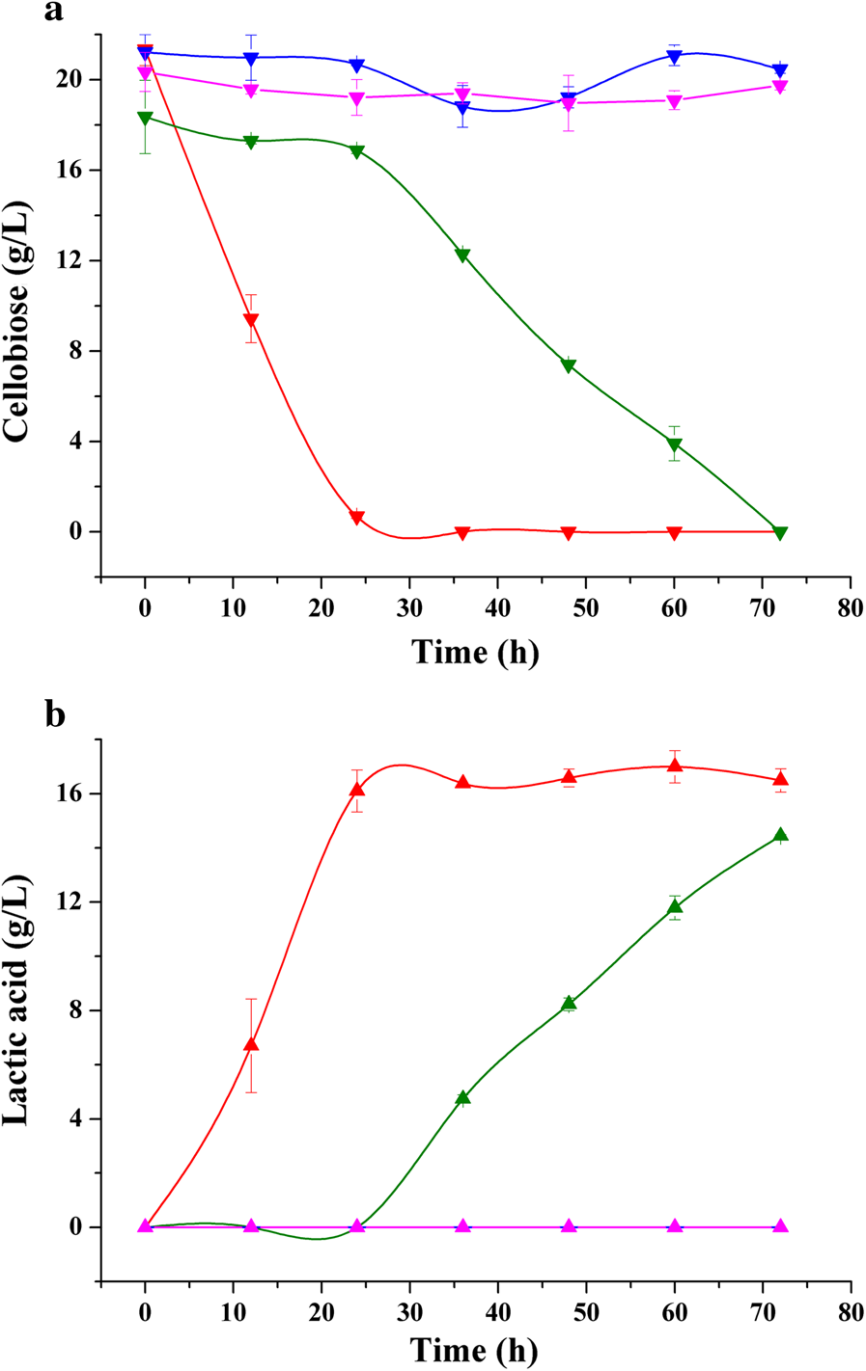
Cellobiose fermentation by *B. coagulans* NL01, ATCC 7050, XZL4, and ATCC 7050(Δ*celR*). **a** Time courses of cellobiose consumption. **b** Time courses of lactic acid production. Red, *B. coagulans* NL01; Olive, *B. coagulans* ATCC 7050; Blue, *B. coagulans* XZL4; Magenta, *B. coagulans* ATCC 7050(Δ*celR*). Fermentations were carried out in 250-mL Erlenmeyer flasks containing 100 mL medium at 50 °C and 150 rpm on a rotary shaker. The medium contained the following (g/L): cellobiose 20, yeast extract 2.5, corn syrup powder 1.2, MgSO_4_·7H_2_O 0.4, (NH_4_)_2_SO_4_ 3, KH_2_PO_4_ 0.22, MnSO_4_·H_2_O 0.03, FeSO_4_·H_2_O 0.03, and CaCO_3_ 10

Next, strains NL01, ATCC 7050 and XZL4 were selected to determine whether the difference in the cellobiose operons affected their cellobiose fermentation ability. Figure 5 shows that strains NL01 and ATCC 7050 could utilize cellobiose, whereas XZL4 could not. Specifically, the utilization rate of cellobiose by NL01 was found to be much more rapid than that of ATCC 7050. Through comparison among NL01, ATCC 7050 and XZL4, it was clear that the above cellobiose operons were crucial to the cellobiose fermentation ability of *B. coagulans* strains. In addition, the CELO2 also seems to play a vital role in enhancing cellobiose metabolism, in spite of this operon lacking the component annotated as the 6-phospho-β-glucosidase encoding gene. Therefore, CELO2 might be an incomplete operon, which hastens the transportation and phosphorylation of cellobiose into cells, but lacks the ability to hydrolyse cellobiose-6-phosphate. Another possibility is that the DUF871 domain-containing protein is a novel 6-phospho-β-glucosidase, and CELO2 plays a stronger role in cellobiose uptake when in vivo.

To clarify the specific role of CELO1 and CELO2 in vivo, different mutants of *B. coagulans* NL01 lacking CELO1, CELO2, and both CELO1 and CELO2 should be constructed as a prerequisite. However, *B. coagulans* was reported to be recalcitrant to genetic engineering [20, 21]. Up to now, genetic manipulation of most *B. coagulans* strains was currently unable to be performed, except *B. coagulans* ATCC 7050 and *B. coagulans* P4-102B [20, 21, 33]. Compared with *B. coagulans* P4-102B, *B. coagulans* ATCC 7050 is a standard and genome-sequenced strain and has been knocked out by several different research groups. Therefore, only the CELO1 mutant derived from *B. coagulans* ATCC 7050 can presently be constructed in our lab. It is significant, however, that the PEP-PTS components of CELO1 from *B. coagulans* NL01 and *B. coagulans* ATCC 7050 share a high level of identity, although two extra transposase genes exist in ATCC 7050. The protein sequences of IIA, IIB, IIC, 6-phospho-β-glucosidase, and the transcriptional regulator from *B. coagulans* NL01 were found to be 96%, 96%, 99%, 96%, and 94% homologous with those from *B. coagulans* ATCC 7050, respectively. Given these facts, the CELO1 mutant of *B. coagulans* ATCC 7050 was used as an alternative for further studying the function of *B. coagulans*’ cellobiose operon in vivo.

Because CELO1 in *B. coagulans* ATCC 7050 is composed of five different and long components (up to 10,397 bp), full knockout of CELO1 was very difficult. Instead, only *celR* was selected as a knockout target to obtain a mutant strain of *B. coagulans* ATCC 7050 (Δ*celR*). Analogously, fermentation of cellobiose by this mutant was carried out and the results are shown in Fig. 5. The fermentation profile of *B. coagulans* ATCC 7050 (Δ*celR*) was similar to that of *B. coagulans* XZL4, which indicates that the incomplete CELO1 lost the function of cellobiose utilization by partial gene knockout. In NCBI, *celR* of *B. coagulans* is annotated as a PRD-containing protein, which means that this protein contains a PTS regulation domain (PRD). So far, the PRD-containing transcription regulators are divided into two groups: antiterminators and transcription activators, and the latter can be further divided into two classes, NifA/NtrC- and DeoR-like PRD-containing transcription activators [34]. In some sequenced bacterial genomes, many transcription activators are incorrectly referred to as antiterminators [34]. This is also the case in *B. coagulans* genomes. The PRDs of *B. coagulans* should be DeoR-like because the molecular weight of different types of PRD-containing transcription regulators is notably different: 30 kDa for antiterminators, 65 kDa for DeoR-like, and 90–100 kDa for NifA/NtrC-like transcription activators [34]. The result of *B. coagulans* ATCC 7050 (Δ*celR*) in Fig. 5 also certifies that *celR* is an indispensable component for CELO1 to perform its function.

**Fig. 6:**
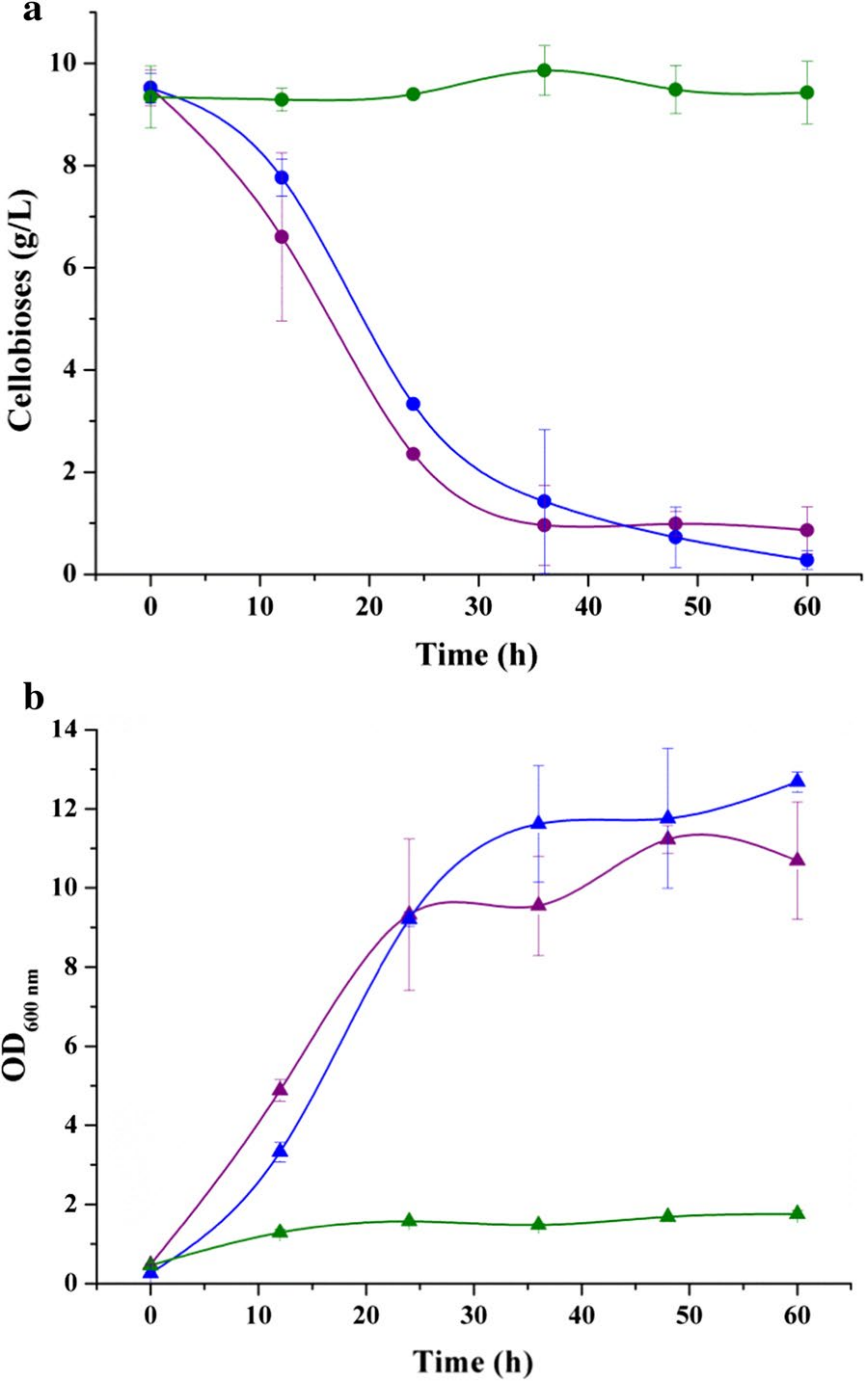
Cellobiose fermentation of *E. coli* BL21(pET-*celo1*), *E. coli* BL21(pET-*celo2*) and *E. coli* BL21(pETDuet-1). **a** Time courses of cellobiose consumption. **b** Time course of optical density. Purple, *E. coli* BL21(pET-*celo1*); Blue, *E. coli* BL21(pET-*celo2*); Olive, *E. coli* BL21(pETDuet-1). Fermentations were carried out in 100-mL Erlenmeyer flasks containing 30-mL LB medium in the presence of 10 g/L cellobiose, 100 mg/L ampicillin, and 0.2 mM IPTG at 37 °C and 200 rpm on a rotary shaker

Although the CELO1 and CELO2 of *B. coagulans* NL01 could not be mutated in vivo, they could be cloned and expressed in heterologous hosts. *E. coli* was reported to be without cellobiose consumption ability except for when it was cultivated using cellobiose as the sole carbon source for a long time. This observation was believed to be due to the cryptic nature of the *bgl* and *cel* operons [35, 36]. Hence, two recombinant strains, *E. coli* BL21(pET-*celo1*) and *E. coli* BL21(pET-*celo2*) were constructed to investigate whether CELO1 and CELO2 could enable a genetically modified *E. coli* to ferment cellobiose. An *E. coli* strain bearing an empty plasmid (pETDuet-1) was designated as *E. coli* BL21(pETDuet-1) and used as control. The cellobiose utilizing capacities of the three engineered strains were tested in shaker flasks. Figure 6 showed that both *E. coli* BL21(pET-*celo1*) and *E. coli* BL21(pET-*celo2*) had better cellobiose metabolism than *E. coli* BL21(pETDuet-1). Both *E. coli* BL21(pET-*celo1*) and *E. coli* BL21(pET-*celo2*) consumed almost all added cellobiose, which indicated that CELO2 is a complete operon and may have a great influence on cellobiose catabolism for *B. coagulans* NL01. In contrast, *E. coli* BL21(pETDuet-1) did not show significant cellobiose consumption. Accordingly, the cell growth of *E. coli* BL21(pETDuet-1) was much weaker compared with *E. coli* BL21(pET-*celo1*) and *E. coli* BL21(pET-*celo2*).

Due to the lack of a mature tool for genetic engineering of *B. coagulans* NL01, knockout of CELO1 and CELO2 in NL01 is different to execute now. However, this study has provided a new research field in terms of increasing our understanding of cellobiose metabolism in *B. coagulans*. Continuing the study of CELO2 and DUF871 domain-containing protein in *B. coagulans* NL01 might provide new insights into the diversity of microbial cellobiose utilization mechanisms and the diversity of 6-phospho-β-glucosidase.

## Conclusions

In this study, a wild type of *B. coagulans* (NL01) was found to employ cellobiose-specific PEP-PTS for translocation and phosphorylation of cellobiose, and intracellular hydrolysis of cellobiose-6-phosphate. With the aid of this unique strategy for intracellular assimilation of cellobiose, co-fermentation of cellobiose and xylose was successfully performed to circumvent glucose repression. Moreover, two different cellobiose operons were identified by comparative genomic analysis and the role of CELO1 and CELO2 was certified, respectively. Overall, *B. coagulans* NL01 would be recognized as a robust microorganism for a biorefinery process and not be limited to lactic acid production. The cellobiose operon described herein can be a promising prototype for development of alternative approach aimed at circumventing the sophisticated glucose repression.

## Additional file

**Additional file 1: Table S1.** The primers used for RT-PCR.

## Abbreviations

SSF: simultaneous saccharification and fermentation
LB: Luria Broth
RT-PCR: reverse transcription PCR
PEP-PTS: phosphoenolpyruvate-dependent phosphotransferase system
CELO1: cellobiose operon 1
CELO2: cellobiose operon 2
*pbgl*: 6-phospho-β-glucosidase gene
PRD: PTS regulation domain
