# Supplementary material for: Simultaneous consumption of cellobiose and xylose by Bacillus coagulans to circumvent glucose repression and identification of its cellobiose-assimilating operons
Source: Biotechnol Biofuels. 2018 Dec 1;11:320. doi: 10.1186/s13068-018-1323-5 (PMC6271610; doi:10.1186/s13068-018-1323-5)
Supplement: Supplementary file 1 — Additional file 1: Table S1. The primers used for RT-PCR. [file 13068_2018_1323_MOESM1_ESM.docx]

**Additional file**

**Simultaneous consumption of cellobiose and xylose by *Bacillus coagulans* to circumvent glucose repression and identification of its cellobiose-assimilating operons**

Zhaojuan Zheng^1, 2#^, Ting Jiang^1, 2#^, Lihua Zou^1, 2^, Shuiping Ouyang^1, 2^, Jie Zhou^1, 2^, Xi Lin^1, 2^, Qin He^1, 2^, Limin Wang^3^, Bo Yu^3^, Haijun Xu^1, 2^, Jia Ouyang^1, 2*^

^1^Jiangsu Co-Innovation Center of Efficient Processing and Utilization of Forest Resources, Nanjing Forestry University, Nanjing 210037, People’s Republic of China

^2^College of Chemical Engineering, Nanjing Forestry University, Nanjing 210037, People’s Republic of China

^3^CAS Key Laboratory of Microbial Physiological and Metabolic Engineering, Institute of Microbiology, Chinese Academy of Sciences, Beijing 100101, People’s Republic of China

^#^These authors contributed equally to this work.

^*^Corresponding author. Address: College of Chemical Engineering, Nanjing Forestry University, Nanjing 210037, People’s Republic of China, Tel.: 86-025-85427129, Fax: 86-025-85427587, E-mail: [hgouyj@njfu.edu.cn](mailto:hgouyj@njfu.edu.cn).

Table S1 The primers used for RT-PCR.

| Primer | Sequence (5′-3′) | Use |
| --- | --- | --- |
| CELO1 |  |  |
| *celA-celB*.f | GAATGCCATGACAGTAATTGACCTGGC | Amplification of overlapping regions spanning the *celA-celB* genes (forward) |
| *celA-celB*.r | ATTGCCTTCATTTGCTGAAACAGCATCGAC | Amplification of overlapping regions spanning the *celA-celB* genes (reverse) |
| *celB-hyp*.f | GCCACAAGCCTATATTCCGATTCCAATGGG | Amplification of overlapping regions spanning the *celB*-*hyp* genes (forward) |
| *celB-hyp*.r | CGGTTATAGTACATCGATTTAAGCATCTGG | Amplification of overlapping regions spanning the *celB*-*hyp* genes (reverse) |
| *hyp-celC*.f | CTGTTTTGATTGCCGGGATATTTTTAAGCGC | Amplification of overlapping regions spanning the *hyp-celC* genes (forward) |
| *hyp-celC*.r | CGATTGTAAATGGTATTGAAGCCATCCCGG | Amplification of overlapping regions spanning the *hyp-celC* genes (reverse) |
| *celC-pbgl*.f | CAAGAAAAAGGCGGAGAATCTGTACTCTGA | Amplification of overlapping regions spanning the *celC-pbgl* genes (forward) |
| *celC-pbgl*.r | CCCCATAAAAAGTTTTCCGGTAAAACGCCT | Amplification of overlapping regions spanning the *celC-pbgl* genes (reverse) |
| CELO2 |  |  |
| *celB-celA*.f | GGCCCGCAAGTCCGTTATATGAAAAAGAAA | Amplification of overlapping regions spanning the *celB-celA* genes (forward) |
| *celB-celA*.r | GCAATTCCTGATCCGCCTCTTCCAGCTTTT | Amplification of overlapping regions spanning the *celB-celA* genes (reverse) |
| *celA-celX*.f | CCGGCCAAACGATTTTCGTATCACTTCTTA | Amplification of overlapping regions spanning the *celA-celX* genes (forward) |
| *celA-celX*.r | TATTCCCCTGAATTTCCAGCAAACTTGCAA | Amplification of overlapping regions spanning the *celA-celX* genes (reverse) |
| *celX-celC*.f | TATTCCCCTGAATTTCCAGCAAACTTGCAA | Amplification of overlapping regions spanning the *celX-celC* genes (forward) |
| *celX-celC*.r | GATTGCCCAGTATGAGAAAAAAAGAACCGA | Amplification of overlapping regions spanning the *celX-celC* genes (reverse) |
